# An Internet-Based HIV Self-Testing Program to Increase HIV Testing Uptake Among Men Who Have Sex With Men in Brazil: Descriptive Cross-Sectional Analysis

**DOI:** 10.2196/14145

**Published:** 2019-08-01

**Authors:** Raquel Brandini De Boni, Valdilea Gonçalves Veloso, Nilo Martinez Fernandes, Flavia Lessa, Renato Girade Corrêa, Renato De Souza Lima, Marly Cruz, Juliane Oliveira, Simone Muniz Nogueira, Beto de Jesus, Toni Reis, Nena Lentini, Raquel Lima Miranda, Trista Bingham, Cheryl C Johnson, Aristides Barbosa Junior, Beatriz Grinsztejn

**Affiliations:** 1 Instituto Nacional de Infectologia Evandro Chagas Oswaldo Cruz Foundation Rio de Janeiro Brazil; 2 National Department of STI, AIDS, and Viral Hepatitis Ministry of Health Brailia Brazil; 3 Escola Nacional de Saúde Pública Oswaldo Cruz Foundation Rio de Janeiro Brazil; 4 Secretaria Municipal de Saúde Curitiba Curitiba Brazil; 5 Grupo Dignidade Curitiba Brazil; 6 Centers for Disease Control and Prevention-Brazil Brasilia Brazil; 7 Division of Global HIV & TB Center for Global Health Centers for Disease Control and Prevention Atlanta, GA United States; 8 HIV Department World Health Organization Geneva Switzerland

**Keywords:** HIV/AIDS, HIV self-testing, key populations, mobile health, men

## Abstract

**Background:**

Approximately 30% of people living with HIV worldwide are estimated to be unaware of their infection. HIV self-testing (HIVST) is a strategy recommended by the World Health Organization to increase access to and uptake of testing among key populations who are at high risk for HIV infection.

**Objective:**

This study aimed to describe the development and feasibility of a free, anonymous, internet-based HIVST strategy designed for men who have sex with men in Curitiba, Brazil (electronic testing [e-testing]).

**Methods:**

The project was developed under the scope of the “A Hora é Agora” (The Time is Now) program. Individuals aiming to request an HIVST package (two tests each) answered an anonymous 5-minute questionnaire regarding inclusion criteria and sexual risk behavior. Eligible individuals could receive one package every 6 months for free. Website analytics, response to online questionnaires, package distribution, and return of test results were monitored via a platform-integrated system.

**Results:**

Between February 2015 and January 2016, the website documented 17,786 unique visitors and 3218 completed online questionnaires. Most individuals self-reported being white (77.0%), young (median age: 25 years, interquartile range: 22-31 years), educated (87.3% completed secondary education or more), and previously tested for HIV (62.5%). Overall, 2526 HIVST packages were delivered; of those, 542 (21.4%) reported a result online or by mail (23 reactive and 11 invalid). During the study period, 37 individuals who reported using e-testing visited the prespecified health facility for confirmatory testing (30 positive, 7 negative).

**Conclusions:**

E-testing proved highly feasible and acceptable in this study, thus supporting scale-up to additional centers for men who have sex with men in Brazil.

## Introduction

In 2016, it was estimated that 30% of people living with HIV worldwide were unaware of their infection [1]. This gap represents a major challenge to achieving the targets of the Joint United Nations Programme on HIV/AIDS (UNAIDS) “90-90-90,” which aims to ensure that 90% of all people living with HIV are aware of their status by 2020 [2].

Unknown HIV status is associated with late entry into care and increased mortality rates and is a key driver of the epidemic [3-5]. An HIV test result is the first step in engaging in the HIV treatment continuum for those who test positive or in the HIV prevention continuum for those who test negative. Nonetheless, many barriers to expand HIV testing coverage persist, such as stigma, difficulty in accessing testing facilities, and inconvenient clinic hours [6,7].

HIV self-testing (HIVST) allows individuals to perform an HIV test and interpret their own results [8]. Different strategies for HIVST distribution worldwide have been proposed, such as through peers [9], nongovernment organizations (NGOs) [10], and mobile/social media apps [11]. Randomized controlled trials have shown that HIVST increases uptake and frequency of HIV testing overall, without adverse events, social harm, or increased risk behaviors [12]. Nevertheless, a review of HIVST implementation studies by Estem et al [13] concluded that despite the many potential benefits of HIVST, dissemination strategies need to be improved and the high cost of HIVST kits needs to be reduced to scale-up this strategy.

Key populations, including gay and other men who have sex with men (MSM), are greatly affected by HIV in Latin America and other regions [14,15]. Such populations have continued high HIV incidence and encounter multiple structural barriers, including stigma and discrimination, which increase their risk of HIV exposure and acquisition and inhibit access to evidence-based HIV prevention and treatment services [16]. In Brazil, the prevalence of HIV among MSM, estimated at 14% in 2009 [17] and 17.5% in 2016 [18], requires sustained prevention and treatment efforts. Furthermore, despite an increased proportion of MSM reporting annual testing (from 21.2% in 2009 to 43.3% in 2016) [19], innovations are needed to increase MSM testing coverage in the country. The convenience and discreet approach of self-testing would be particularly useful to increase first-time HIV testing and frequency among this vulnerable population.

Internet-based and mobile phone technologies are promising tools to overcome HIVST delivery barriers among MSM [20]. In the United Kingdom and United States, both online and social media–based promotion and delivery of free HIVST kits effectively increased the frequency of testing and reached MSM who were not previously tested [21,22]. In accordance with international data [23,24], a previous Brazilian study suggested that HIVST would be well accepted among MSM [25]. Thus, providing HIVST using these technologies may represent a step forward in increasing testing access in Brazil, where free HIV testing is already provided by the Brazilian Public Health System (“Sistema Unico de Saúde”).

Considering the limited access to and uptake of HIV testing among MSM in Brazil and the evidence supporting its acceptability, this paper reports on the development and feasibility of an internet-based HIVST (electronic testing [e-testing]) approach. E-testing was designed to promote HIV prevention by providing free anonymous HIVST and to enhance linkage to HIV care for those with a confirmed HIV positive status. We describe the results from the e-testing program’s first year, when HIVST was not available in Brazil outside the research studies.

## Methods

### Design

This cross-sectional analysis describes the results obtained from the e-testing strategy between February 2015 and January 2016.

E-testing was developed as part of the broader initiative “A Hora é Agora” (AHA; or “The Time is Now”), which included three community-based strategies to deliver rapid HIV testing: a mobile testing unit, an NGO site, and e-testing. AHA was implemented in partnership with the Oswaldo Cruz Foundation (FIOCRUZ); the Department of STI, AIDS, and Viral Hepatitis of the Brazilian Ministry of Health; the Municipal Health Secretariat of Curitiba; the Federal University of Paraná; Grupo Dignidade (a local Lesbian Gay Bisexual Transgender and Queer/Questioning NGO); and Centers for Disease Control and Prevention (CDC) - Brazil. Implementation of the AHA program was focused in Curitiba, a city with 1.75 million inhabitants in southern Brazil and an estimated HIV prevalence of 19.9% (95% CI 14.2-27.2) among MSM [18].

### Recruitment

The AHA program counted on an extensive communications plan to increase HIV testing and target young MSM [26]. The appropriateness of the communications strategy was piloted in focus groups including the target population, conducted in the preformative research (which also mapped the main MSM gathering sites and possible community partners to the program). The communication plan was continuously monitored and evaluated during the entire program via independent focus groups conducted in 2016 and Google Analytics.

**Figure 1:**
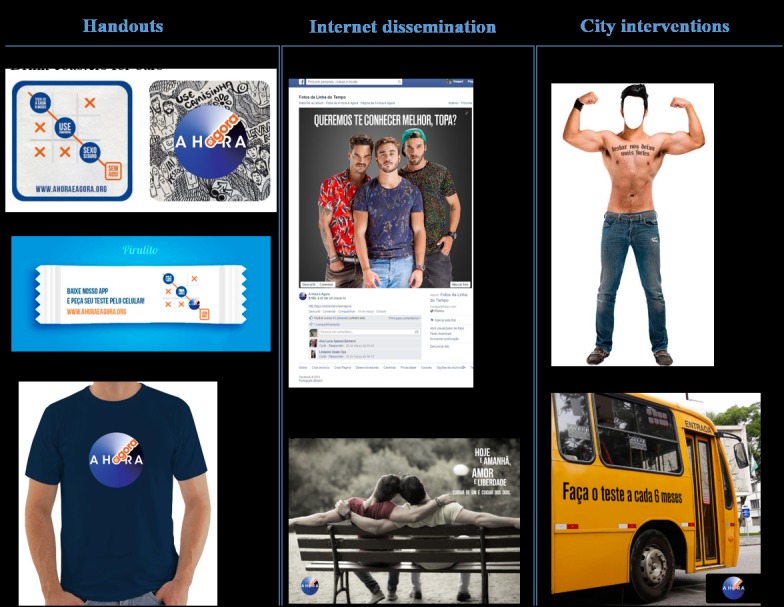
Sample of the “A Hora é Agora” communications strategy for Curitiba, Brazil (2015-2016).

Briefly, the communications included printed handouts distributed via in-person outreach events in places where MSM socialize in the city; partnerships with gay and MSM-friendly establishments such as saunas, movie theatres, cafes, and bars; and virtual messages disseminated trough a Facebook page and gay online sites such as ManHunt and Grindr. Examples of the materials used for AHA are presented in Figure 1.

### Study Population

Participants were eligible to receive an HIVST package if they were male at birth, were at least 18 years of age, resided in Curitiba, were HIV-negative or had an unknown HIV status, had access to the internet, and agreed to participate in the study after reading the online informed consent form.

### E-Testing Web Platform

**Figure 2:**
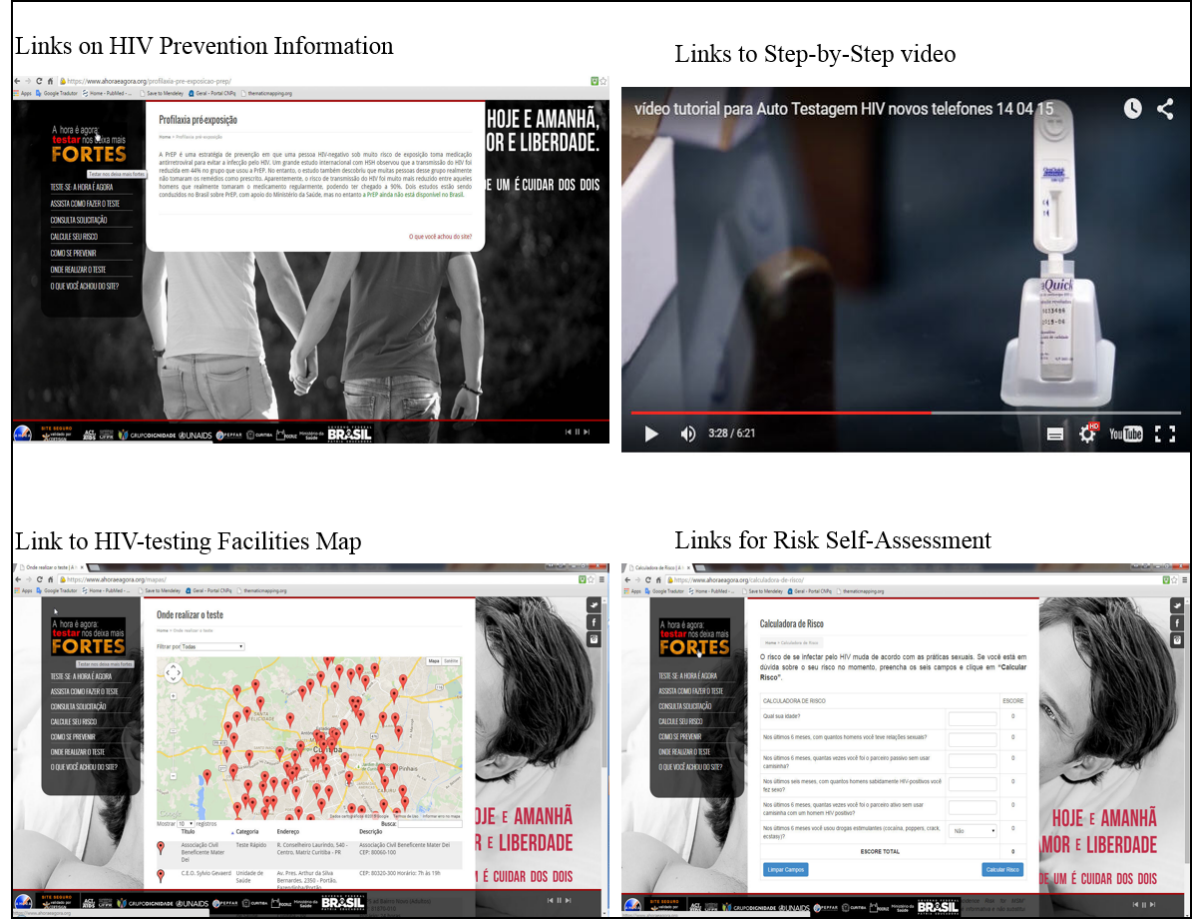
E-testing Web platform: general information module.

The internet platform was made available to all at www.ahoraeagora.org and could be accessed via personal computer, smartphones, or tablets (apps were available for IOS and Android). This platform contained four modules (ie, website sections presenting different access credentials): general information, HIVST order, management, and monitoring. The general information module contained HIV prevention information targeted to MSM, a step-by-step video, written instructions for using HIVST, geocoded options for HIV testing locations in Curitiba, and a personal risk assessment calculator (Figure 2).

**Figure 3:**
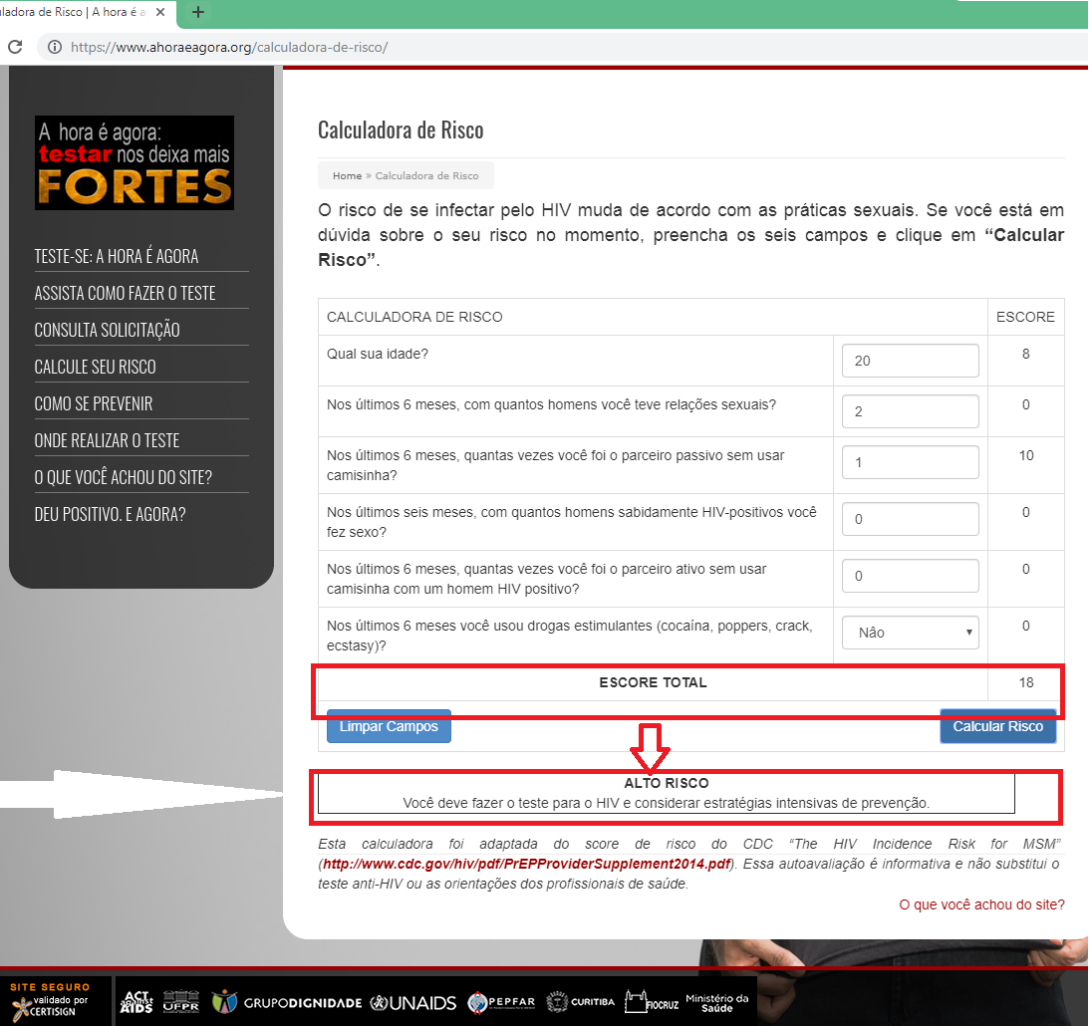
E-testing Web platform: risk assessment calculator.

The risk assessment calculator was adapted from the HIV incidence risk for MSM scale, a 7-item questionnaire developed to predict HIV seroconversion among MSM [27] and recommended by the CDC to screen individuals for eligibility for pre-exposure prophylaxis [28]. Individuals could check their risk status and receive feedback, but data were not collected/saved in the platform. (Figure 3).

The HIVST order module included an online questionnaire and the option to request HIVST. This module evaluated eligibility criteria and enabled test kit delivery via standard Brazilian mail or self-pick up at a central pharmacy within 2 weeks. After completing the mandatory section of the questionnaire and selecting a delivery option, a random personal identification number (PIN) was assigned by the automated system. The PIN was used to track future HIVST requests, collect self-reported HIVST results, and monitor subsequent linkage to further testing and HIV care.

The management module encompassed the administration and logistics functions of HIVST package delivery (including central and pharmacy inventory control) and generated management reports for authorized staff. Finally, the fourth module monitored access and usability statistics of the AHA website (using Google Analytics), aggregated data from the order module, and generated routine reports.

### HIV Self-Testing Package

MSM who met the eligibility criteria and accessed the e-testing website could order an HIVST package. The package included two OraQuick Advanced HIV-1/2 HIVST kits, (OraSure Technologies, Inc, Bethlehem, PA, adapted and repackaged for self-testing in Brazil), condoms, lubricants, written self-test instruction, an anonymous prepaid card for returning test results (for those preferring to return the results by mail), and information about health facilities that provided free confirmatory HIV testing. Between March 7, 2015, and May 4, 2015, and between August 14, 2015, and February 29, 2016, only one HIVST was sent in each package. Using their PIN, individuals could order a new package every 6 months.

### Measures

#### Website Analytics, HIV Self-Testing Distribution, and Platform Usability

Website analytics were measured using Google Analytics. Data on HIVST distribution were retrieved from the management module. Platform usability was measured using the following questions:

- Question: “Overall, did you find this platform easy to use?” Response: Yes/No
- Question: “What were the major difficulties you find in the platform?” Responses: “I was not able to find the links I was looking for” / “I would like to have access to more tests” / “I would like to retrieve the tests in other places” / “I did not find any difficulties” / “Other-specify”
- Question: “Did you find the testing instructions clear?” Response: Yes/No.

#### Characteristics of the Study Population

Individuals requesting an HIVST package were encouraged to answer the online questionnaire available in the HIVST order module. The questionnaire comprised two parts: the first was mandatory to initiate the HIVST request and included the eligibility criteria (eg, participant’s age, city, and unknown or negative HIV status). The second part was optional and evaluated demographic characteristics (eg, schooling, color/race, gender and sexual orientation, and steady partner), risk perception (using the question “What is your likelihood of getting HIV in the next 12 months?”) [29], and risk behavior for HIV infection (using the HIV incidence risk for the MSM scale) [27].

#### HIV Self-Testing Result Return

Individuals were asked to return their results either by directly entering result information into the website (using their PINs) or via the prepaid postal card. Additionally, when uploading results to the website, individuals were invited to answer a questionnaire regarding their experience and any possible social harm (using three dichotomous questions regarding coercion, feelings of shame, or violent reactions of partners).

#### Confirmatory Testing

Individuals with a reactive or invalid test result were instructed to seek confirmatory testing at Curitiba’s reference center for HIV Counseling and Testing (COA) and to bring their PIN. The COA follows the HIV testing algorithm of the 2014 Brazilian Ministry of Health guidelines [30], and those confirmed to be HIV-positive were offered immediate HIV care. A question regarding the AHA testing strategies was included in the regular COA form, and staff reported the number of tests performed as a result of referrals from specific AHA project strategies.

#### Feasibility Outcomes

Project success was defined *a priori* as ≥60% individuals ordering HIVST after starting the online request process; ≥50% individuals picking up the HIVST at the pharmacy within 2 weeks; 1000 HIVST packages (2000 HIVST kits) distributed in 12 months; 20% HIVST results returned via mail or the online platform; and ≥50% individuals, who self-reported a reactive HIVST, accessing the COA for confirmatory testing and HIV care. There were no *a priori* outcomes related to the second HIVST kit included in the package.

Although there was scarce evidence to determine all the feasibility outcomes at the time of the study design (2014), a limited-advertised Brazilian Web survey was able to reach 629 MSM in around 10 days, of whom 82 were from the South region of the country. The vast majority (89.8%) of them reported high interest in using HIVST [25]. As the South of the country has three state capitals, and this project had a strong communications plan, we expected to distribute around 80 HIVST per month (rounded to 1000/year) in the selected South Capital (Curitiba). For the outcomes that we had no previous data on, we considered the most conservative estimate (50%) to be acceptable. Finally, regarding return of the testing results, we considered the general low response of mail and email surveys conducted in different areas of knowledge [31-34] and the additional absence of incentives; as such, we expected that 20% of the results would be returned to the project.

### Statistical Analysis

We present the results of the online questionnaires and HIVST results returned using descriptive statistics, including both absolute counts and relative frequencies and 95% CIs. Data were analyzed using SPSS software (IBM Corp, Armonk, NY).

### Ethical Issues

Details of informed consent were displayed in pop-up windows preceding the online questionnaire. No personally identifying information was collected in the informed consent forms or questionnaires to preserve users’ anonymity.

Personal information necessary for HIVST delivery was saved in the Web server and was restricted to trained staff in charge of package mail delivery. All staff members signed a confidentiality agreement. Identification for HIVST delivery was not linked to individuals’ questionnaire responses. To ensure information security and confidentiality, the website used verified access to the original site, data encryption between personal computers and the website, and encryption of database tables. Information was only available to users registered with a login and a safe password.

Trained counselors at a 24/7 hotline were available to assist in the event of any psychological crisis. Social harm, such as coercion or violent reactions of partners, was considered an adverse event and was assessed in the feedback questionnaire.

The e-testing study protocol was approved by the Evandro Chagas National Institute of Infectious Diseases/FIOCRUZ Institutional Review Board (CAAE number: 37848114.9.0000.5262). The study was reviewed according to the Centers for Disease Control and Prevention human research protection procedures and was determined to be research but the CDC was not engaged.

## Results

**Figure 4:**
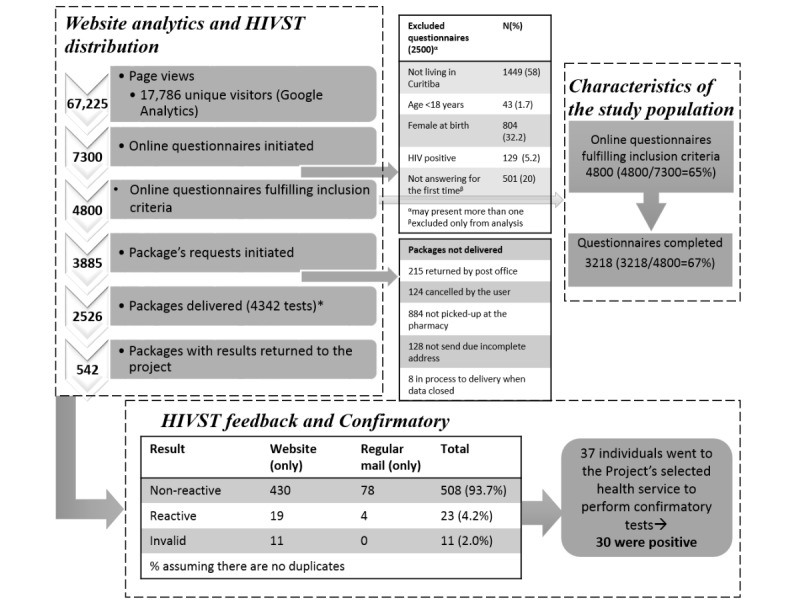
E-testing project flowchart for Curitiba, Brazil (2015-2016). *Between March 7, 2015, and May 4, 2015, and between August 14, 2015, and February 29, 2016, only one HIVST was sent in each package; therefore, the total number of unique HIVST kits distributed is 4342. HIVST: HIV self-testing.

The detailed frequencies and data sources of the e-testing study during its first year of implementation are provided in Figure 4.

### Website Analytics, HIV Self-Testing Distribution, and Platform Usability

During the study period, the AHA website had 67,225 page views from 17,786 unique visitors. The most frequently viewed pages were those in which users could order an HIVST package by mail (9.7%), all pages containing information on HIV prevention (6.6%), and the page on risk self-assessment (6.5%; Table 1). The e-testing app had 2571 downloads (1780 downloads at Google Play and 791 at Apple Store).

Overall, 3885 questionnaire respondents initiated an HIVST request, of which 2526 packages were delivered (215 requests were returned by mail, 124 were cancelled by the users, 884 were not picked up at the pharmacy within 2 weeks, 128 were not sent due to incomplete addresses, and 8 were in the process of delivery at the time the present dataset was closed; Figure 4). Most deliveries were made by the Brazilian mail (n=1943, 77%), and 583 (23%) packages were picked up at the pharmacy. A total of 66 PINs (of 3885, 1.7%) requested at least one additional HIVST package during the study period.

Of all individuals accessing the website, 362 answered questions about the usability of the website. Most respondents found the site “Very easy” (n=260, 71.8%) or “Easy” (n=71, 19.6%) to use. Furthermore, 72% (262/362) stated that they did not have difficulties navigating the website, and only 21 (5.8%) stated that they did not find the pages they were searching for. The vast majority (n=339, 93.6%) also found that the website’s instructions for performing HIVST were clear.

**Table 1:** Pages viewed (n=67,225) on the A Hora é Agora e-testing website in Curitiba, Brazil (February 6, 2015, to January 31, 2016).

Pages viewed		Value, n (%)^a^
**Any of the following links on HIV prevention information**		4425 (6.6)
	Viral load and treatment as prevention	337 (0.5)
	Circumcision	750 (1.1)
	Sexual transmitted diseases	280 (0.4)
	Interrupted intercourse	746 (1.1)
	Pre-exposure prophylaxis	418 (0.6)
	Postexposure prophylaxis	583 (0.9)
	Oral sex	946 (1.4)
	Condom use	365 (0.5)
Link for risk self-assessment		4398 (6.5)
Link to HIV-testing facilities map		3420 (5.1)
Link to HIVST^b^ request by mail		6546 (9.7)
Link to pick up HIVST in the pharmacy		1917 (2.9)
Link to step-by-step video		4118 (6.1)

^a^Proportion of hits in a specific link over total hits for the website.

^b^HIVST: HIV self-testing.

### Characteristics of the Study Population

During the study period, 65% (4800/7300) of individuals who initiated an HIVST request met the eligibility criteria. Of the men who completed the online questionnaire (n=3218), most were young (median: 25 years; interquartile range [IQR]: 22-31 years), were of white race/ethnicity (n=2478, 77%), had completed at least secondary school (n=2810, 87%), and had never had an HIV test (n=1206, 37.5%; Table 2). The frequency of possible reasons for never testing were “I am afraid of having a positive result” (27.6%), “I feel ashamed” (21.7%), “It is inconvenient to go to a health care clinic” (15.7%), “I am not at risk of being infected” (10.4%), and “I feel lazy” (7.4%); 17.3% did not want to answer this question.

**Table 2:** Demographic and behavioral characteristics of men who completed the online questionnaire at the A Hora é Agora e-testing website in Curitiba, Brazil 2015-2016 (N=3218).

Characteristic			Value, n (%)
**Demographics**			
	Age (years), median (IQR^a^)		25 (22-31)
	**Race/ethnicity**		
		White	2478 (77.0)
		Black	103 (3.2)
		Mixed	495 (15.4)
		Native	22 (0.7)
		Asian	49 (1.5)
		Don’t know/don’t want to answer	71 (2.2)
	**Schooling**		
		No schooling/incomplete primary school	85 (2.6)
		Complete primary/incomplete secondary school	261 (8.1)
		Complete secondary/incomplete college	1549 (48.1)
		Complete college/graduate/professional school	1261 (39.2)
		Don’t know/don’t want to answer	62 (1.9)
	**Sexual identity/orientation**		
		Gay/homosexual	1953 (60.7)
		Bisexual	370 (11.5)
		Heterosexual	649 (20.2)
		Transvestite, transsexual, transgender	22 (0.7)
		Other	25 (0.8)
		Don’t know/don’t want to answer	199 (6.2)
	**Steady partner**		
		Yes	1334 (41.5)
		No	1677 (52.1)
		Don’t want to answer	207 (6.4)
**Risk perception**			
	**Perceived likelihood of acquiring HIV in the next 12 months**		
		None/little chance	1973 (61.3)
		Some chance/high chance/certainly	266 (8.3)
		Don’t want to answer	978 (30.4)
	**Had a previous HIV test in lifetime**		
		Yes	2012 (62.5)
		No	1206 (37.5)
**Risk behavior for HIV infection**			
	**Number of male sexual partners (6 months)**		
		0-5	1486 (46.2)
		6-10	345 (10.7)
		>10	197 (6.1)
		Don’t know/don’t want to answer	1190 (37.0)
	**Number of times participant was receptive to partner without condom (6 months)**		
		None	14 (0.4)
		Once or more	1097 (34.1)
		Don’t know/don’t want to answer	2107 (65.5)
	**Number HIV-positive partners (6 months)**		
		None	34 (1.0)
		1	231 (7.2)
		>1	37 (1.2)
		Don’t know/don’t want to answer	2916 (90.6)
	**Number of times participant had condomless insertive anal sex with HIV-positive partner (6 months)**		
		0-4	419 (13.0)
		≥5	149 (4.7)
		Don’t know/don’t want to answer	2650 (82.3)
	**Stimulant use (6 months)^**b**^**		
		Yes	525 (16.3)
		No	2466 (76.6)
		Don’t know/don’t want to answer	227 (7.1)
	**STI^c^** **diagnosis in prior 6 months**		
		Yes	249 (7.8)
		No	2728 (84.7)
		Don’t know/don’t want to answer	241 (7.5)

^a^IQR, interquartile range.

^b^Stimulants include cocaine, crack, poppers, and ecstasy.

^c^STI: sexually transmitted infection.

### Return of HIV Self-Testing Results

Of the 2526 packages delivered, 21.4% (n=542) returned an HIVST result on the website or by mail. Of these, 19 reactive and 11 invalid test results were entered into the website platform and 4 reactive results were returned by mail (as of February 29, 2016).

There were 91 responses to the feedback survey on self-testing, but we received no reports (questionnaire or hotline) of psychological crises or violent reactions from partners. However, one individual reported feeling ashamed because someone saw him self-testing.

### Confirmatory Testing

As of February 2016, 37 individuals were tested at the COA reporting to have used the e-testing: 30 had HIV-positive results and 7 had negative results.

### Feasibility Outcomes

Project success was achieved for most of the previously defined outcomes (Table 3).

Over the study period, the telephone hotline received 70 calls: two were questions regarding what to do following a reactive result, three were related to assistance with interpreting the self-test result, five were related to queries on test performance, six were related to problems with HIVST delivery, and the remaining were about HIV testing in general and not about self-testing/e-testing.

**Table 3:** Feasibility outcomes defined in the study protocol and achievements in the A Hora é Agora e-testing project in Curitiba, Brazil (2015-2016).

Measurement	Proportion	Indicator	*A priori* success indicator
Individuals who got an HIVST^a^ package after starting the online request process	2526/3885	65.0%	≥60%
Individuals who collected the HIVST at the distribution pharmacy, within 2 weeks of ordering	544/1417	38.4%	≥50%
HIVST packages distributed during the initial 12-month timeframe (either sent by mail or retrieved at the pharmacy)^b^	Not applicable	2526^c^	≥1000^c^
Proportion of HIVST packages with a test result returned^d^ via mail or the website up to February 29, 2016	(82^e^+460^f^)/2526	21.4%	≥20%
Proportion of those with a positive result who accessed the Counseling and Testing Center for confirmatory testing up to February 29, 2016	30^g^/34^h^	88.2%	≥50%

^a^HIVST: HIV self-testing.

^b^Between March 7, 2015, and May 4, 2015, and between August 14, 2015, and February 29, 2016, only one HIVST was sent in each package; therefore, the total number of unique HIVST kits distributed is 4342.

^c^The N value is presented.

^d^Different date (February 29, 2016) was considered in this indicator to provide enough time for individuals to receive/retrieve tests and include the results on the platform.

^e^Overall, there were 90 results returned by mail, but 8 were also included on the site (n=82 test results returned by mail). Four unique reactive results were returned by mail.

^f^460 test results returned on the website.

^g^Overall, 37 individuals went to Counseling and Testing Center to access confirmatory testing: 30 were positive and 7 were negative. Given the absence of identification, we cannot confirm that these are the same individuals who reported their results into the site or by mail.

^h^19 reactive+11 invalid inserted in the platform+4 reactive returned by mail up to February 29, 2016.

## Discussion

We describe an internet-based strategy to provide HIV prevention information and HIVST to MSM in Curitiba, Brazil. In the first year, we delivered 2526 HIVST packages, substantially more than predicted in the study design (1000 packages). Our findings are similar to reports from previous studies that have shown good uptake and use of websites containing information on HIV prevention, self-assessment of risk, and HIVST among MSM [35,36]. Such high uptake is likely due to the significant number of MSM who use social media and the internet to look for sexual partners [37]. A previous Web-based survey in 10 Brazilian cities [38] found that 1798 (35.6%) and 678 (13.4%) of responding MSM reported daily and weekend use of websites and smart phone apps to seek sexual partners.

The use of internet and social media approaches for health promotion and behavioral interventions has become more routine according to international literature and already includes smoking cessation [39] and heart disease prevention programs [40]. The internet and social media are particularly promising ways to offer testing to MSM at high ongoing risk of HIV [41], as those using social media and the internet to meet sexual partners also have high-risk behavior and increased HIV risk [42,43]. Thus, internet-based health initiatives can help overcome health disparities to access prevention and care, particularly among MSM, and can help facilitate uptake of HIV testing, prevention, and treatment services.

E-testing appeared to appeal to never-tested MSM who reported a fear of having a positive result, shame (which is probably related to stigma), and unfriendly health facilities. These results are consistent with reports showing that HIVST is highly acceptable to MSM [25,44] and can facilitate uptake and frequency of testing. This demand was likely driven by the privacy of e-testing, which systematic reviews have highlighted as a priority for MSM [23]. Similarly, the large number of HIVST packages delivered by mail, compared to packages retrieved in the public pharmacy, was likely related to introducing delivery models that addressed challenges such as travel distances, wait times at facilities, and confidentiality issues [45,46], all of which have been previously cited as barriers to testing.

Of the individuals who answered the entire online questionnaire, most identified as young, white, well-educated, and without a steady partner. Although these characteristics were similar to those reported in other studies among MSM in the country [38,47], there were some differences as compared to a recent household probability survey conducted among men from Curitiba [48]: In our study, participants were younger, more likely to be white (77% vs 64%), less likely to perceive high risk for HIV infection (8% vs 52%), and less likely to have ever had an HIV test (62.5% vs 75.7%) than found in the survey [48].

However, the latest Brazilian estimates have shown an increase in the HIV prevalence among young MSM [18], who also showed riskier sexual behavior than older MSM [19]. This new evidence reinforces the importance of strategies to successfully reach this key population. Our findings suggest that, at least for young MSM, important subpopulations are open to new HIV testing strategies and understand the need for frequent or risk-based HIV testing.

In our study, HIVST was provided for free but without the provision of any incentives (the Brazilian Ethic regulations do not allow the provision of incentives for research participants or patients). We received 21.4% of HIVST results (18.2% through the website and 3.2% by mail). This was a substantially lower proportion than presented in China by Zhong et al [49], who reported 97% of self-testers returning results when HIVST kit costs were refunded upon return of results. We believe that this difference is related to the provision of incentives, and not providing incentives was one of the reasons why we estimated a low return rate when designing this feasibility study. However, the World Health Organization recommends self-testing as an additional HIV testing tool [8], not as an HIV surveillance tool, meaning that a low return rate may not represent a major drawback. On the other hand, confirmatory testing and rapid linkage to care are essential after a reactive HIVST result. A study conducted in Brazil reported that of 131 MSM who tested positive, only 95 (72.5%) were linked to care [50]. Because of the anonymity of the e-testing strategy, the proportion of study participants who sought confirmatory testing is uncertain. Furthermore, although users were instructed to bring their PIN to the COA when seeking confirmatory testing, no one accessing services at COA provided a PIN. Therefore, we could not match the HIVST results and confirmatory results. Other programs seeking ways to track users’ confirmatory testing and linkage to HIV services following HIVST should be aware of this limitation and the challenges with monitoring HIVST implementation. Other potential caveats of self-testing discussed in the literature are psychological crises and social harm. In our study, neither of these events were reported, which is consistent with the international literature [12,23].

E-testing was largely successful during its first year: E-testing achieved most of the previously defined study outcomes, and demand for HIVST was twice that initially anticipated. However, our study had limitations. The anonymous, self-reported nature of data collection and the low proportion of users who reported their HIVST results prevented us from validating individual HIVST results with confirmatory tests and from estimating the HIV prevalence (due to the inability to exclude multiple answers from the same individuals). Additionally, because we did not track the second test distributed in the package, it is possible that the test requestor gave the second test to a friend or partner, which would increase the number of individuals tested during the study period. We likely also missed MSM who may be fearful of HIV testing and those who did not have access to the internet, which limits the generalizability of our findings. Despite these limitations, we achieved our primary goal, which was to reach individuals who do not access traditional HIV testing services.

In conclusion, our findings show that an internet-based strategy to provide free, anonymous HIVST kits was acceptable and feasible in Brazil. Lessons learned from this study may also be applied to other countries in Latin America with similar cultural and epidemiological contexts. The discretion and convenience of HIVST may increase testing among MSM who are not reached through existing services. Future investigation is needed, however, to identify strategies to support linkage to prevention and treatment services and to determine the potential public health impact and cost-effectiveness of HIVST in Brazil.

## Abbreviations

AHA: “A Hora é Agora” (The Time is Now) program
CDC: Centers for Disease Control and Prevention
COA: HIV Counseling and Testing Center
FIOCRUZ: Oswaldo Cruz Foundation
HIVST: HIV self-testing
IQR: interquartile range
MSM: men who have sex with men
NGO: nongovernmental organization
PEPFAR: President’s Emergency Plan for AIDS Relief
PIN: personal identification number
STI: sexually transmitted infection
UNAIDS: Joint United Nations Programme on HIV/AIDS
WHO: World Health Organization
